# Phase Change Materials Composite Based on Hybrid Aerogel with Anisotropic Microstructure

**DOI:** 10.3390/ma14040777

**Published:** 2021-02-07

**Authors:** Chen Li, Dong Zhang, Wanwan Ren

**Affiliations:** Key laboratory of Advanced Civil Engineering Materials, Ministry of Education, School of Materials Science and Engineering, Tongji University, Shanghai 200092, China; a15826123644@163.com (C.L.); wanwanren@tongji.edu.cn (W.R.)

**Keywords:** phase change materials, graphene, aerogel, thermal conductivity, thermal storage performance

## Abstract

Phase change materials (PCMs) can be thermally enhanced by reduced graphene oxide (rGO)/expanded graphite (EG) aerogel with anisotropic microstructure. An rGO/EG aerogel with anisotropic microstructure was prepared by directionally freezing aqueous suspensions of graphene oxide (GO) and EG, followed by a freeze-drying process and thermal reduction at 250 °C. The anisotropic microstructure of rGO/EG aerogel composite PCM was confirmed by scanning electron microscopy (SEM), thermal conductivity tests and infrared images. The thermal conductivity of PCMs increased remarkably with rGO/EG aerogel. Compared with the thermal conductivity of pure paraffin, it increased by about 50~300% in the longitudinal direction and increased by about 25–150% in the transversal direction. The enhancement of thermal conductivity was attributed to the improvement of the thermal pathway provided by rGO/EG aerogel and the decrease of the interfacial thermal resistance between PCM and fillers. Meanwhile, rGO/EG aerogel was combined with paraffin only by physical adsorption, and no chemical interaction occurs between them, leading to no effect on the phase change behavior. In addition, the addition of rGO/EG aerogel led to a slight increase in the latent heat of the paraffin in the composite PCM.

## 1. Introduction

Alternative energy sources, such as solar energy, are more and more adopted to solve problems caused by the consumption of fossil fuels [1]. Among these substitutes, the emergence of thermal energy storage systems has attracted much attention. Because it not only helps to reduce the dependence on fossil fuels but also contributes to the efficient and benign use of energy. Similarly, phase change materials (PCMs) also has caught attention in the field of thermal energy storage [2,3] and temperature control [4,5] because of their high-energy density, stable chemical properties, non-corrosive, nontoxicity, low price and repeatable utilization [6,7,8]. However, the major weakness of PCMs is their low thermal conductivity [9,10]. To improve this weakness, thermally conductive fillers, such as metallic and carbon-based materials [11], including mental foam [12], β-aluminum nitride powder [13], carbon nanotubes [14,15,16], graphite powder [17] and expanded graphite (EG) [18], are chosen to enhance the thermal conductivity of composite PCM.

Thermally conductive fillers are usually added to PCMs by means of mechanical blending. Although this method is very convenient, agglomeration and precipitation of fillers are apt to occur after multiple phase transitions. These problems can be avoided by three-dimensional (3D) thermal conductive fillers [19], which own shape stability during phase transition and can improve the thermal conductivity meanwhile retaining high latent heat retention. In recent years, many efforts have been made in 3D thermal conductive filler materials. Hong et al. [20] made a 3D polypropylene reinforced phase change composite with thermal conductivity of 0.534 W∙m^−1^∙K^−1^, which was twice that of pure paraffin. Xiao et al. [12] prepared paraffin/nickel foam composite PCM. The measurement results show that the thermal conductivity of paraffin containing nickel foam is about three times higher than that of pristine paraffin. Obviously, a well-arranged thermally conductive network can provide a more efficient phonon transmission path and will not deposit during the phase transition from these studies. In addition, PCMs with the anisotropic 3D thermal conductive fillers have excellent thermal conductivity for the heat transfer path along a specific direction and decreasing interfacial resistance between the PCMs and the fillers. Li Qi et al. [21] reported that through the manufacture of multilayer graphene that is vertically aligned and densely packed in the epoxy matrix, the thermal conductivity of the multilayer graphene/epoxy composite has been significantly improved. Haiyan et al. [22] found that magnetic field action during curing significantly improved the thermal conductivity of epoxy resin/Fe_3_O_4_ modified graphene composites. Compared with epoxy resin/non-directional graphene composites, the thermal conductivity of epoxy resin/directional graphene composites prepared under an external magnetic field increased by 41%.

In previous studies, graphene oxide (GO) aerogels can significantly improve the electrical properties [11] and thermal properties of composites. Hydrothermal method [23], auxiliary drying method [11] and directional freezing method are generally used in the preparation of aerogel loaded with graphene oxide. Lee et al. [24] reported treating GO with melamine resin (MR) monomers to avoid severe restacking between graphene individual sheets, resulting in the carbonaceous composite with an exceptionally large surface area of 1040 m^2^/g. Highly thermally conductive and porous expanded graphite (EG) is well suited for increasing the thermal conductivity of PCMs and adsorbing PCMs. The amphiphilic structure of GO makes hydrophobic EG evenly dispersed in GO solution. Ren et al. [23] prepared an isotropic rGO/EG aerogel composite PCMs by hydrothermal method, whose thermal conductivity is enhanced equally in all directions. Moreover, graphene sheets can be readily fabricated into anisotropic 3D interconnecting networks by directional freezing [25], magnetic-induced method [26], layer-by-layer assembly method [25], in situ polymerization method [27], etc. However, anisotropic aerogels consisting of graphene and EG have not been reported.

Herein, anisotropic reduced graphene oxide (rGO)/EG aerogels are fabricated by directionally freezing aqueous suspensions of GO and EG, followed by freeze-drying process and thermal reduction at 250 °C. After impregnation with paraffin, the resulting composite PCM exhibits anisotropic thermal conductivity. Anisotropic aerogel networks could provide more efficient thermal transfer pathways and decrease thermal resistance. The rGO/EG aerogels account for only 3–9% of the mass of the composite PCM, but the longitudinal thermal conductivity of phase change composites is greatly increased. When rGO/EG aerogel content is 8.7 wt.%, the longitudinal thermal conductivity of composite PCM increase to 0.79 W·m^−1^·K ^−1^, almost 4 times of pure paraffin (0.19 W·m^−1^·K ^−1^). Compared with the experimental results of Ren et al. [23], we can find that the anisotropic phase change composite materials of the same composition have higher thermal conductivity and have the function of directional heat conduction. At the same time, the low proportion of rGO/EG aerogel also guarantees the energy storage capacity of the composite PCM.

## 2. Experiment

### 2.1. Materials

EG and the graphite flakes powder (200 meshes) were purchased from Baoding Lianxing Cemented Carbide Co., Ltd. (Baoding, China). Sulfuric acid (H_2_SO_4_, 98%), potassium permanganate (KMnO_4_, 99.9%), hydrochloric acid (HCl, 37%), hydrogen peroxide (H_2_O_2_, 30%), and paraffin were purchased from Sinopharm Chemical Reagent Co., Ltd. (Shanghai, China).

### 2.2. Preparation of rGO/EG Aerogel

**Figure 1:**
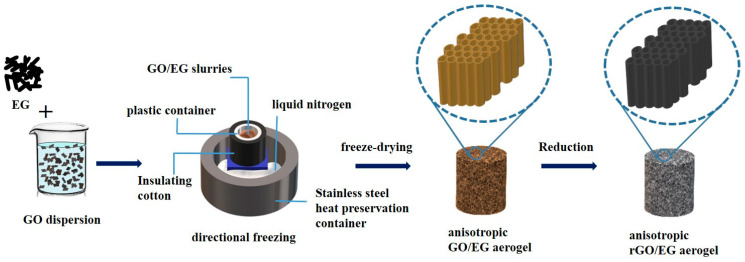
Flow chart of anisotropic reduced graphene oxide (rGO)/expanded graphite (EG) aerogel produced by directional freezing.

GO was prepared by the modified Hummers method [28]. First, 80 mL of 10 mg/mL GO aqueous was dispersed evenly in a beaker, and the mass ratio of EG in the GO/EG mixture is 20%, 40%, 60%, and 80%. Second, the EG of the corresponding mass was weighed into GO aqueous dispersion and stirred for 30 min. The GO/EG slurries were frozen directionally by liquid nitrogen. In the process of directional freezing, liquid nitrogen was placed in the stainless steel insulation container, and the stainless steel support was placed in the liquid nitrogen so that the surface of the liquid nitrogen and the upper surface of the support was flush. The GO/EG slurries were placed in the plastic container on the stainless steel support and wrapped with insulating cotton. After freezing, the samples were freeze-dried for 96 h to obtain the anisotropic GO/EG aerogel. Lastly, the GO/EG aerogel was cut into pieces with a thickness of about 1 to 2 cm in the transversal or longitudinal direction for reduction. When reducing the sample, the samples were placed on an iron rack in the vacuum drying oven; the oven was turned on to heat it. The timer was started after the temperature reached 250 °C. Samples were heated under vacuum at 250 °C for two hours. The preparation flow chart of rGO/EG aerogel is shown in Figure 1.

### 2.3. Preparation of Composite PCM

The composite PCM was prepared by paraffin and rGO/EG aerogel. The rGO/EG aerogel was cut into a round cake of 1 to 2 cm in diameter and 2.5 mm in thickness. The rGO/EG aerogel was immersed in the melted paraffin for 1 min to completely absorb the paraffin. The aerogel slowly sank after gradually absorbing the paraffin but did not collapse. The rGO/EG aerogel composite PCM was obtained after paraffin cooling and solidification.

### 2.4. Characterization

The morphology of the samples was investigated by scanning electron microscope (Jeol, Tokyo, Japan). The chemical structure was measured by Fourier-transform infrared (FTIR, Bruker EQUINOXSS) (Bruker, Madison, WI, USA) and X-ray photoelectron spectroscopy (XPS, PHI-500 °C ESCA) (Thermo Elemental, Waltham, MA, USA), the X-ray source power was set to 150 W, and the incident photon energy was 20 eV. Raman spectra were obtained from a Raman microprobe (H Evolution, Horiba Jobin Yvon, Paris, France). The phase transition properties of the samples were characterized by a differential scanning calorimeter (DSC, TA Instruments, Q100) (TA Instruments, Delaware, DE, USA). An infrared thermal imager (FLIR, ETS320) (FLIR Systems, OR, USA) was used to observe the temperature variation of the samples during temperature changes. The temperature was measured by a thermocouple (CENTER-309) (Center Technology, New Taipei City, Taiwan). Thermal conductivity λ (W∙m^−1^∙K^−1^) was measured using the steady-state plate method [29]. Surface area and porosity characterization were obtained by N_2_ adsorption–desorption at 77 K with NOVA 2200e surface area and a pore size analyzer.

## 3. Results and Discussion

### 3.1. Microstructure and Chemical Structure of rGO/EG Aerogel

**Figure 2:**
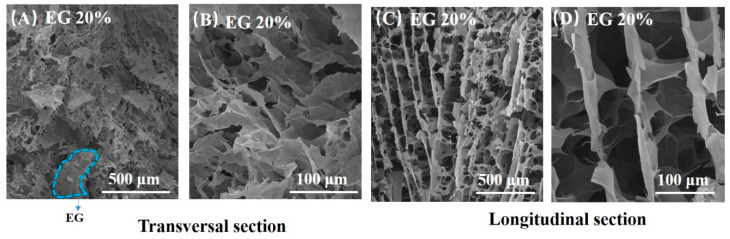
SEM photos of transversal section of rGO/EG aerogel with EG content of 20% (**A**,**B**), SEM photos of longitudinal section rGO/EG aerogel with EG content of 20% (**C**,**D**).

Figure 2 displays SEM photos of rGO/EG aerogel with EG content of 20%. Many rGO sheets can be observed from the SEM photos, and the rGO sheets show an orderly arrangement to some extent. Especially in the longitudinal section SEM photos, it can be seen that rGO sheets grow vertically and appear to be fence-like. Aligned rGO sheets are connected by shorter rGO sheets. This is due to the directional freezing with gradient temperature distribution. When the sample was directionally frozen, the ice crystals grew in an orderly upward direction along the temperature gradient. Because GO sheets have good hydrophilicity, they adhered easily to the ice template. After freeze-drying and reduction, the rGO sheets exhibited the structure of the original ice template, which had ordered fence shape. Hence, rGO/EG aerogel exhibited an aligned microstructure.

**Figure 3:**
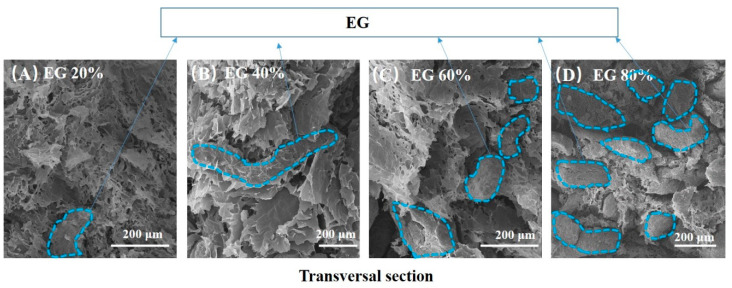
SEM photos of the transversal section of anisotropic rGO/EG aerogel with EG content of 20% (**A**), SEM photos of the transversal section of anisotropic rGO/EG aerogel with EG content of 40% (**B**), SEM photos of the transversal section of anisotropic rGO/EG aerogel with EG content of 60% (**C**), SEM photos of the transversal section of anisotropic rGO/EG aerogel with EG content of 80% (**D**).

Figure 3 displays SEM photos of the transversal section of rGO/EG aerogel with increasing EG. As we can see, EG appears worm-like, and rGO is sheet-like from Figure 3. The dashed blue line circles part of EG. The more EG that was added, the greater the proportion of worm-like objects appeared, as shown in Figure 3A,D. The GO and EG always showed good uniformity no matter how much EG was added. Moreover, the sheet-like graphene covered the expanded graphite. At the same time, the sheet-like graphene overlapped with each other to form a three-dimensional structure. It could be clearly observed that the overlapping graphene EG was embedded in the sheet. This shows that the combination of EG and graphene sheet is relatively uniform, and the graphene sheet constituted a bridge between the EG, which was beneficial to improve the thermal conductivity. At the same time, it can be seen that the three-dimensional structure contained a large number of voids, which was beneficial for the aerogel material to adsorb more PCMs.

**Figure 4:**
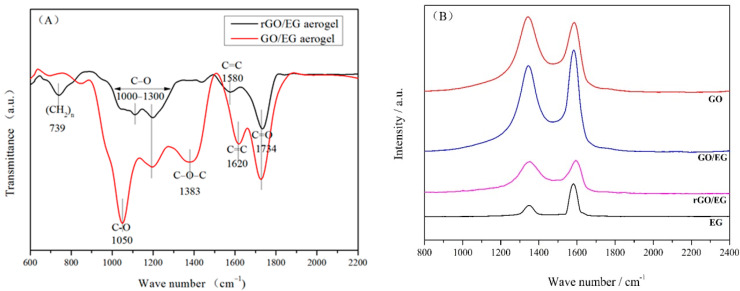

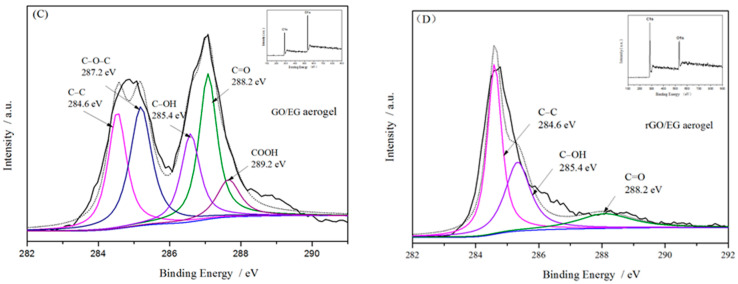
FTIR spectra of GO/EG aerogel and rGO/EG aerogel (**A**), Raman spectra of expanded graphite (EG), graphene oxide (GO), GO/EG aerogel and rGO/EG aerogel (**B**), XPS of GO/EG aerogel and rGO/EG aerogel (**C**,**D**), The upper right corner picture shows the ratio of carbon to oxygen of GO/EG aerogel and rGO/EG aerogel (**C**,**D**).

Figure 4A shows the FTIR spectra of GO/EG aerogel and rGO/EG aerogel. Peaks at 1050, 1000 to 1300 and 1734 cm^−1^ in the FTIR spectrum of GO/EG aerogel correspond to the vibrations of C–O, C–O–C, and C=O [30]. The peak at 1383 cm^−1^ indicates the deformation vibration of the C–O–C group [30]. GO/EG aerogel had a significant peak at 1620 cm^−1^, which was the skeleton vibration of C=C. The double bond vibration in rGO/EG aerogel almost disappeared, but the peak of the benzene ring skeleton appeared at 1580 cm^−1^, and the peak of the long-chain carbon chain appeared at 739 cm^−1^. The structure and properties of the hydrocarbons changed from hydrophilic to hydrophobic. The double bonds transformed to benzene rings and single bonds, which also proved that the sample did undergo a reduction reaction. Compared to GO/EG aerogel, the peaks in the FTIR spectrum of rGO/EG aerogels were significantly reduced but did not completely disappear. This indicated that a fraction of the oxygen-containing functional groups still existed in the rGO/EG aerogel.

Figure 4B shows the Raman spectra of EG, GO, GO/EG aerogel and rGO/EG aerogel. From the picture, we can see that the peaks at 1350 and 1596 cm^−1^ in the Raman spectrum of carbon material correspond to the vibrations of the D bond and G bond, respectively. The G band was produced by the stretching motion of all sp^2^ atomic pairs in the carbon ring or long-chain. Defects and disorder in carbon atoms induced the D band [31]. Generally, the intensity ratio of D band to G band (I_D_/I_G_) indicated the disorder degree of the carbon materials [32]. The I_D_/I_G_ of EG, GO, GO/EG aerogel, and rGO/EG aerogel were 0.38, 1.04, 0.89, 0.95, respectively. The oxygen-containing functional groups on the GO were the main reasons for the high I_D_/I_G_ of GO, compared to EG. Although most oxygen-containing functional groups were removed after thermal reduction, I_D_/I_G_ of GO/EG increased from 0.89 to 0.95 of rGO/EG instead of decreasing, which means that the thermal reduction process caused additional lattice distortion in the aerogel.

Figure 4C,D shows the XPS spectra of GO/EG aerogel and rGO/EG aerogel. In the spectrum of GO/EG aerogel, the binding energy peaks of 284.6 eV, 287.2 eV, 285.4 eV, 288.2 eV, and 289.2 eV represent the five chemical groups of C–C, C–O–C, C–OH, C=O and COOH, respectively [33,34]. In the spectrum of rGO/EG aerogel, the peak of C–O–C and COOH binding energy almost disappears, the peaks of C–OH and C=O binding energy cannot be ignored, indicating that there was still a part of oxygen-containing groups present on the carbon chain structure. On the other hand, the peak value of the binding energy of C–C was greatly increased, indicating that the functional group of rGO/EG aerogel existed in a branched form and did not affect the main chain structure of the carbon chain. At the same time, the atomic ratio of C/O could also quantitatively characterize the reduction effect. After reduction, the C/O atomic ratio of the sample increased from 2.125 to 4.0. This aspect reflects most of the oxygen-containing groups were reduced, which was consistent with FTIR characterization.

**Figure 5:**
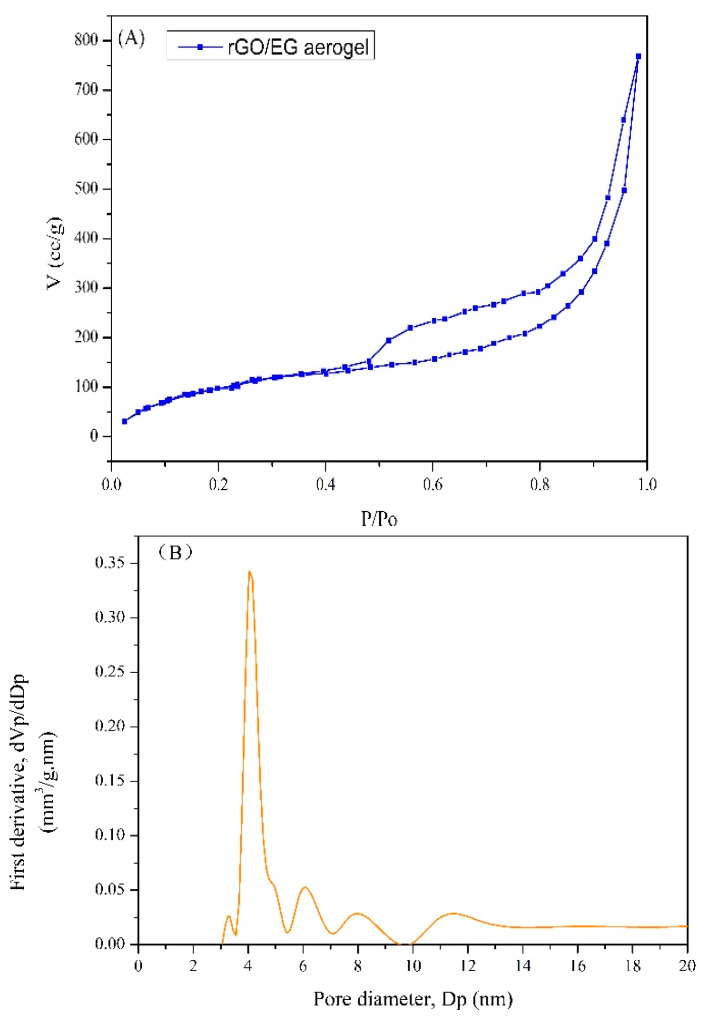
N_2_ adsorption–desorption isotherm of rGO/EG aerogel (**A**), Barrett–Joyner–Halenda (BJH) pore size distribution of rGO/EG aerogel (**B**).

The N_2_ adsorption–desorption isotherms for rGO/EG aerogel with EG content of 80% is shown in Figure 5. The BET surface area of rGO/EG aerogel was 420 m^2^/g, which was a big improvement over previous graphene aerogels with a surface area of 199.3 m^2^/g [35]. A multimodal pore size distribution (Barrett–Joyner–Halenda (BJH)) pore distribution) was centered at 4.1, 6.1, 7.9 and 11.5 nm. Most of the pores in rGO/EG aerogel were mesoporous.

### 3.2. Microstructure and Chemical Structure of Composite PCM

**Figure 6:**
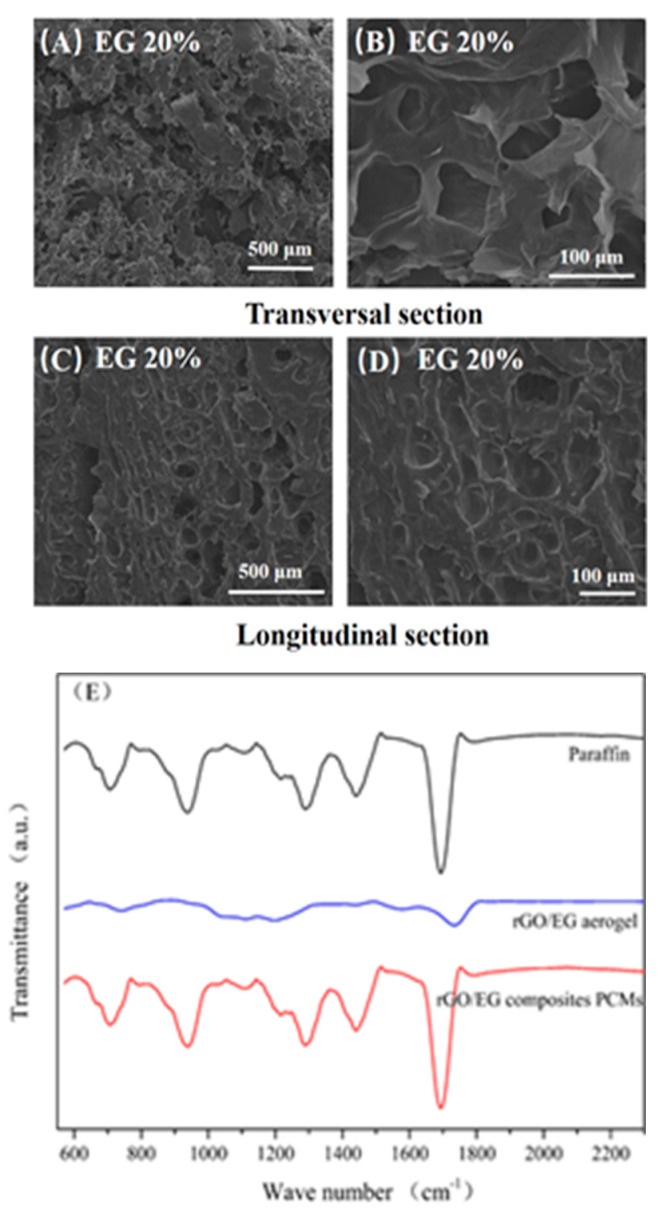
SEM photos of composite phase change materials (PCM) (**A**–**D**) and FTIR spectra of rGO/EG aerogel, paraffin and composite PCM (**E**).

Figure 6A–D displays SEM image of rGO/EG aerogel composite PCM with EG content of 20%. It could be observed that the composite PCM section was smooth, indicating that the paraffin adsorption was relatively sufficient. A similar structure could be observed by comparing the SEM photos of the aerogel. In the transversal sectional image, the upright rGO sheets could be seen and completely covered with paraffin wax; the flat areas were large pieces of EG, which become smooth after adsorption of paraffin. In the longitudinal sectional image, a shallow fence type structure could be observed, and EG was attached thereto. This shows that the 3D structure of the aerogel, especially the anisotropic properties, was preserved during the adsorption of paraffin.

Figure 6E shows the FTIR spectra of paraffin, rGO/EG aerogels, and composite PCM. The main peaks of paraffin were 705 cm^−1^, 938 cm^−1^, 1285 cm^−1^, 1443 cm^−1^ and 1692 cm^−1^, which were related to the vibration of –CH_2_, C–O, C–O–C, –CH_3_ and C=O groups [33]. In the FTIR spectra of composite PCM, the peak structure was almost the same as paraffin, and there was no other obvious peak and significant peak shift. On one hand, it showed that aerogels account for very little in the composite PCM, and their absorption peaks were almost not reflected in the infrared spectrum. On the other hand, there was no obvious peak shift, indicating that there was no obvious chemical bond between the aerogels and paraffin. Aerogels were combined with paraffin only by physical adsorption, and no chemical reaction occurs. Consistent with SEM analysis, the structure of aerogel did not change when compounded with paraffin.

### 3.3. Phase Change Properties of Composite PCM

**Figure 7:**
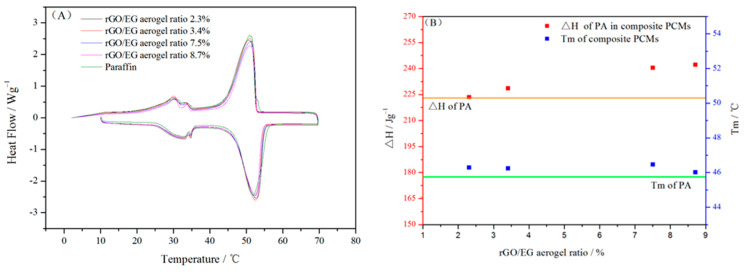
Differential scanning calorimeter (DSC) curves of paraffin and composite PCM (**A**); phase change properties of paraffin in composite PCM (**B**).

Figure 7A shows the DSC heating and cooling curves of composite PCM and paraffin. The latent heat and melting temperature of composite PCM and paraffin were calculated from DSC curves in Figure 7A and shown in Figure 7B. It can be seen from the line chart that the melting temperature (Tm) of pure paraffin was 45.75 °C Tm of the composite PCM was slightly higher by 1–2 °C than that of paraffin. This indicates that the rGO/EG aerogel with a 3D structure did not have a major impact on the phase transition process of paraffin. The latent heat of paraffin in composite PCM was calculated as follows:

(1) $$∆H=\frac{\mathrm{the}\;\mathrm{average}\;\mathrm{of}\;∆\mathrm{Hm}\;\mathrm{and}\;∆\mathrm{Hc}\;\mathrm{of}\;\mathrm{composite}\;\mathrm{PCM}}{\mathrm{the}\;\mathrm{mass}\;\mathrm{ratio}\;\mathrm{of}\;\mathrm{paraffin}\;\mathrm{in}\;\mathrm{composite}\;\mathrm{PCM}}$$

where ΔHm and ΔHc are melting and freezing latent heat, respectively. The latent heat (ΔH) of pure paraffin is 223.5 kJ/kg. Compared with paraffin, the ΔH of paraffin in composite PCM increased by about 1 to 8.4%. There were many reasons that affect the latent heat of paraffin in composite PCMs [23], such as the thermal conductivity, mass and crystallization rate of samples.

### 3.4. Thermal Conductivity of Composite PCM

**Figure 8:**
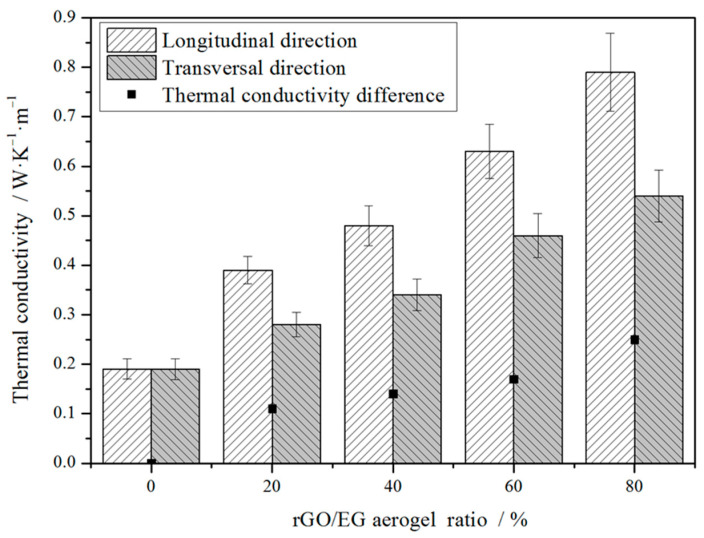
Thermal conductivity of composite PCMs versus rGO/EG aerogel ratios.

Figure 8 shows the thermal conductivity of composite PCM in different directions. Since the sample owns an anisotropic structure, the thermal conductivity varies in different directions. The longitudinal direction was parallel to the direction in which the rGO sheets were arranged. The transversal direction was perpendicular to the direction in which the rGO sheets were arranged. With increasing rGO/EG aerogel ratio, thermal conductivity in the longitudinal direction increased more significantly than that in the transversal direction. It can be seen that the thermal conductivity in the longitudinal direction increased greatly with the increase of the rGO/EG aerogel ratio, most of which was between 0.3 and 0.8 W∙m^−1^∙K^−1^ and the highest set of samples was close to 0.8 W∙m^−1^∙K^−1^. Compared with 0.19 W∙m^−1^∙K^−1^ of pure paraffin, it increased by about 50~300%. However, the thermal conductivity of PCMs in the transversal direction differed from that in the longitudinal direction, which was only about 0.25~0.5 W·m^−1^·K^−1^. In the longitudinal direction, the heat was transmitted along the fence-like rGO sheet with low resistance due to the high thermal conductivity of the rGO. However, in the transversal direction, the lower thermal conductivity along the transverse direction should have been mainly due to the lack of continuous high thermal conductivity pathways formed by rGO. Hence, the anisotropic structure of rGO/EG aerogel had a differential influence on heat conduction. Moreover, the more rGO/EG aerogel was added, the higher the thermal conductivity of the composite PCM and the larger the thermal conductivity difference between the transversal and longitudinal directions.

**Figure 9:**
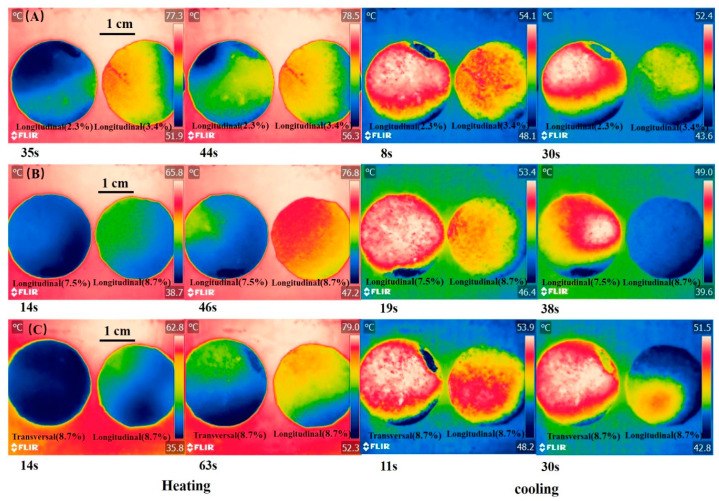
Infrared images of the longitudinal section of composite PCM with 2.3% rGO/EG aerogel and the longitudinal section of composite PCM with 3.4% rGO/EG aerogel during heating and cooling; (**A**) infrared images of the longitudinal section of composite PCM with 7.5% rGO/EG aerogel and the longitudinal section of composite PCM with 8.7% rGO/EG aerogel during heating and cooling; (**B**) infrared images of the transversal section and longitudinal section of composite PCM with 8.7% rGO/EG aerogel during heating and cooling (**C**).

The infrared images of composite PCM during heating and cooling are shown in Figure 9. During heating, the temperature rise rate of the longitudinal section of composite PCM with 3.4% rGO/EG aerogel was faster than that of the longitudinal section of composite PCM with 2.3% rGO/EG aerogel. A similar phenomenon is also observed in Figure 9B. During the cooling process, it was not surprising that the longitudinal section of composite PCM with 3.4% rGO/EG aerogel descends faster than the longitudinal section of composite PCM with 2.3% rGO/EG aerogel. The same findings can be found in Figure 9B. The results show that rGO/EG aerogel could enhance the thermal conductivity of composite PCM again. The longitudinal section of composite PCM with 8.7% rGO/EG aerogel had a faster heating and cooling rate than the transversal section of composite PCM with 8.7% rGO/EG aerogel (shown in Figure 9C). This also confirms that PCM had an anisotropic structure and had a better thermal conductivity in the longitudinal direction than in the transversal direction.

## 4. Conclusions

In this work, we prepared anisotropic rGO/EG aerogel composite PCMs by directional freezing. Moreover, the thermal conductivity of paraffin was greatly improved. Due to the high porosity and high specific surface area of the carbon material aerogel, a fully filled aerogel composite phase change material was obtained after impregnation. Tests consisting of thermal conductivity and infrared thermal imager showed an ideal anisotropic thermal conductivity. Such high thermal conductivity could be attributed to the anisotropic aerogel acting as a thermally conductive skeleton in the phase change material and having an efficient thermal path. In the next future, we will apply these rGO/EG composite PCMs with directional thermal conductivity to construction, temperature control and other fields.

## Data Availability

Data sharing is not applicable to this article.
